# Wheezy Child Program: *The Experience of the Belo Horizonte Pediatric Asthma Management Program*

**DOI:** 10.1097/WOX.0b013e3181c6c8cb

**Published:** 2009-12-15

**Authors:** Laura Lasmar, Maria Jussara Fontes, Maria Teresa Mohallen, Ana Cristina Fonseca, Paulo Camargos

**Affiliations:** 1 Pediatric Pulmonology Unit, University Hospital, and Department of Pediatrics, Medical School, Federal University of Minas Gerais, Belo Horizonte, Brazil; Wheezy Child Program, Municipal Health Authority, Belo Horizonte, Brazil

**Keywords:** asthma program, children, adolescents, health services

## Abstract

**Background:**

Until 1994, assistance provided by the Municipal Public Health System to children with asthma in Belo Horizonte, Brazil, was ineffective because it focused only on treating exacerbations. This scenario motivated the implementation of the Wheezy Child Program.

**Objectives:**

The main objectives were to reduce emergency room visits and hospitalizations.

**Methods:**

The strategies adopted were as follows: continued medical education for pediatricians, reorganization of public assistance into different levels of complexity regarding asthma care, and free dispensation of inhaled beclomethasone, albuterol, and valved spacers. A partnership between the Pediatric Pulmonology staff of the Federal University of Minas Gerais and the Belo Horizonte Municipal Health Authority made these strategies come to fruition, especially through the training of health workers and the devising of protocol after GINA guidelines.

**Results:**

Of 2149 patients with a history of hospitalization after program admission, only 453 were re-admitted in the 6 to 12 months after, a reduction of 79%. There was a 300% increase in the use of aerosol and a reduction to 50% in the use of oxygen-driven nebulizers (*P *< 0.001) in the management of exacerbations. For patients enrolled in the program with moderate and severe persistent asthma, the median adherence rate was 50%.

**Conclusions:**

The Wheezy Child Program has significantly reduced hospitalizations and emergency room visits, has improved quality of life, and has shown that programs of this kind are feasible in low- to middle-income countries. More than 30,000 children have been assisted by the program, and now it seeks to optimize asthma control and increase adherence rates.

## Introduction

Belo Horizonte, a city with 2.4 million inhabitants, is the capital of the state of Minas Gerais. Approximately 60% of the population depends exclusively on the public health system. Before 1994, assistance to children with asthma provided by the Municipal Public Health System in Belo Horizonte, Brazil, was ineffective because it focused only on treating exacerbations. This motivated the implementation of the Wheezy Child Program (WCP), aimed at reducing emergency room visits and hospitalizations. The target population of the WCP is those children and adolescents younger than 15 years.

One of the studies that prompted this project and showed the virtual neglect of children with asthma was conducted in 1994 in the Campos Sales outpatient referral clinic (CSORC) with 178 children and adolescents with asthma who were previously seen in emergency services and hospitals. The results showed that most patients (89.9%) had frequently been in emergency rooms (once or twice a month) and 64% of them had been hospitalized before at least once. Data also showed that the type of care given to the patient in the public system was limited to exacerbations, even though 81% of them had moderate or severe persistent asthma, which requires continuous treatment with inhaled steroids. The assistance was deemed ineffective and of high social cost. The children had no lasting connection with the health team working in primary care facilities because this team was not able, in terms of material and human resources, to attend to the patients' main needs: anti-inflammatory treatment and education [1].

These were the data that the pilot outpatient clinic Campos Sales presented to the health authorities of Belo Horizonte, and coupled with data presented at the IV Municipal Conference of Belo Horizonte in 1993 revealed childhood asthma as a major municipal health issue. Reports of this conference showed that, in that year, there were 2344 hospitalizations because of asthma in children younger than 15 years, a number that could be even higher because children are often admitted with a diagnosis of pneumonia [1].

Hundreds of thousands of dollars were spent in the purchase of aminophylline and short-acting oral *β*-agonists, known to be ineffective in the control of the illness. In the same year, the cost of a single hospitalization was $150 (US dollars), an amount that would cover the annual prophylactic treatment for up to 3 patients. Therefore, public assistance given to children and adolescents with asthma was ill-directed, and despite the availability of human, financial, and material resources, it had little to no clinical and epidemiological impact because they were not properly allocated.

The high-hospitalization and re-admission rates, along with frequent visits to emergency services, showed that these patients were given some public assistance, even if fragmented and of severely limited clinical impact. The nonexistence of a periodic follow-up of children with asthma in the primary health care facilities dislodged the demand to emergency services and hospitals, and it was necessary to direct managing efforts so patients would be linked to primary care--the gateway to the public health system--to be treated [1].

Aiming to change this scenario, the Wheezy Child Program was developed, a joint venture between the Pediatric Pulmonology Unit of the University Hospital (Federal University of Minas Gerais) and the Belo Horizonte Public Health System.

## Program Objectives

The main objectives of the program were (and are) to reduce the main morbidity indicators (emergency room visits and hospitalizations) and to improve the quality of life of children and adolescents with asthma.

## Strategies

The program was organized along 3 axes: (1) continued medical education courses and educational activities for patients and relatives; (2) reorganization of the public assistance given to children with asthma at primary, secondary, and tertiary levels, thus allowing a link between patient and health team in basic units; (3) making inhaled medication and valved spacers available for control of exacerbations and maintenance treatment.

## Program Implantation

The asthma program was implemented in the Municipal Public Health System, that is, 130 primary health care facilities, 4 outpatient referral clinics, and the University Hospital. The existent assistance model provided a satisfactory amount of human, material, and financial resources and an adequate environment for implementing the systematized health activities.

After the first demonstration project, developed in 1994, in the CSORC, the program was implemented progressively, beginning in the second half of 1996 with the Sanitary District where CSORC was located, and directed by a technical cooperation between the Federal University of Minas Gerais (UFMG) and the municipality of Belo Horizonte. The process was installed and sustained by the municipal authorities' political preoccupation in improving the quality of life of children with asthma, a concern held by the successive administrations since 1994 and which adopted as reference the *Global Initiative for Asthma *(GINA) guideline [2].

During this period, key initial assessments were done to adapt the initial strategies. The first one was carried out in the West Sanitary District (WSD), where CSORC, 13 other health centers, 1 emergency service, and 1 district pharmacy are located. The results showed that it was possible to improve the assistance given thereto because there was a marked decrease in hospital re-admissions in the children linked to the primary care facilities, from 21% in 1996 to 8% in 1997 (*P *< 0.001). However, at that time, the WSD results showed no reduction in first admissions (12.8% to 10.8%, *P *= 0.85).

Apart from the regular patient follow-up in the primary care facilities, there was a need to work intensively on the gateway to hospitalization (the emergency services) and increase the program's coverage in the area of each of the 130 primary health care facilities.

In this first assessment 60% of the children included in the WSD pilot project between May 1996 and March 1997 were diagnosed with pneumonia, meaning that the same patient was hospitalized at different times with pneumonia and asthma. All charts were carefully reviewed by one of the authors of the present study (Lasmar), along with epidemiologists of Belo Horizonte Municipal Health Authority. Criteria for diagnosing asthma included a family history of asthma, 3 or more previous wheezing episodes, and unequivocal clinical response to inhaled short-acting *β*_2_-agonists and/or systemic corticosteroids. On the basis of these criteria, 100% of the reviewed medical charts indicated patients with asthma, and therefore, final diagnosis was asthma in the reclassification.

Therefore, the diagnostic workup for new patients should not exclude those previously hospitalized for pneumonia or asthma, and the assistant pediatrician was in charge of differential diagnoses.

The preliminary WSD results were important because they provided issues for later discussion and led to the expansion of the project to the remaining 8 Sanitary Districts. The Municipal Authority health workers and the Pediatric Pulmonology team at UFMG developed a specific set of guidelines, adapted from GINA, with the achieved results. From this aim, the Municipal Health Authorities General Directors decided to prioritize and reorganize the public assistance to children and adolescents with asthma, leading to actions in several operational levels of the asthma program.

Discussion about the changes in the process involving the 9 sanitary districts and other support sectors mobilized the health technicians of the Municipal Health Authority and the Pediatric Pulmonology Unit of the University Hospital (UFMG). Didactic material was created, emphasizing the discussion of the fragmentation of the assistance provided and the role of each category of health professionals involved (pediatricians, nurses, and pharmacists) in the process of providing quality assistance.

Diagnostic workup of patients, that is, children referred by an emergency service or hospital with diagnosis of either asthma or pneumonia, presumed their posterior enrollment in the primary care facility and required work in all levels of the Municipal Public Health System. This team effort was fundamental and initiated a discussion about changing the work process in all sectors, for it was with precise and punctual attention that improvements could be made in terms of the involvement of patients and their families. The differences perceived by the teams during this stage concerned not only the screening and identification of each patient--because he or she already came from another health center and had received vaccinations, regular pediatric care, growth and development assessments, and medications for treating exacerbations--but also a change in the entire asthma treatment regimen.

## Training of the Primary Care Facility Health Team

With a change in perspective, from attention to acute episodes only to antimaintenance treatment, there was a need to focus on capacitating human resources. This responsibility was undertaken by the Pediatric Pulmonology team of University Hospital, along with Belo Horizonte pneumopediatricians.

The main objective of the partnership and the Belo Horizonte Municipality was the training of all 300 pediatricians active in the primary care facilities, and this was to be carried out in the Pediatric Pulmonology Unit at the University Hospital and outpatient referral clinics.

In 1988, the Sanitary Reform Movement led to the creation of SUS, a public health system that replaced the previous assistance model and that focused on curative medicine and had more restricted access. One of the tenets of SUS is to decentralize the health system to redirect resources to municipalities. Thus, the city of Belo Horizonte was able to control and coordinate its own health system, both public and private, allowing for input acquisition and team capacitation. This process made possible the implementation of the Wheezy Child Program.

During the same period, the Department of Pediatrics of the UFMG Medical School, after a pedagogic paradigm shift that focused on both teaching and assistance, and in association with the Belo Horizonte Municipal Authority, carried out team capacitation in some municipal basic health units.

The basic methodology of continued medical education (CME) consisted of periods of concentration (UFMG and CROSC) and dispersion (at primary health centers). The concentration step (theoretical-practical) involved training and lectures that lasted 4 hours for a 4-week period. Using the local guidelines adapted from GINA, its main objective was to update diagnosis criteria and treatment of asthma, bearing in mind integral assistance to the child; also, another objective was to emphasize the need to verify the risks and benefits of therapy with inhaled steroids, as part of a strategy to prioritize inhalation therapy both in acute asthma and in maintenance treatment--along with peak expiratory flow monitoring--and educational activities.

During the dispersion step, the pediatrician acted in his primary care facility and could, therefore, raise new questions for the next period of concentration. A complementary supervised internship (64 hours) was offered to qualify a group of pediatricians in their respective districts.

At the end of the CME, teams of each of the 9 districts could perceive that, in addition to the required theoretical knowledge to properly handle asthma, they had regained their professional self-esteem. The project execution was marked by their high spirits, as they now felt able to intervene and incorporate effective, up-to-date therapeutic resources to their pediatric practice and improve their work process.

Training of the nursing team was carried out in the primary health care facilities and in some referral units by pediatricians and pediatric pulmonologists of the Municipal Authority. Training resources were also created, containing the methodology, based on the discussion of real clinical cases, and emphasizing both their epidemiological and clinical sides, real-world situations reported by the team, the screening mechanism of children with asthma, and instructions for inhalation therapy and peak expiratory flow measurements. Finally, the effort allowed for the discussion of the service organization and the involvement of the health team in outpatient control, where treatment adherence and quality of inhalation technique, among others, would be verified.

From August 1996 to December 1999, the partnership between UFMG and the Municipality of Belo Horizonte trained 250 nursing professionals and 10 pharmacists responsible for the district pharmacies. In 2001 the continued education process expanded to the training of family general practitioners.

## Antiasthmatic Drugs and Valved Spacers

Beclomethasone diproprionate and albuterol--along with plastic valved spacers made in Brazil--were the only 2 asthma drugs provided free to all patients. Medication obtained through public funding was distributed in the district pharmacies and later made available in the primary care facilities.

Initially, in the WSD, imported spacers were used, whose high cost compromised its continuous supply and thus the program was unable to provide these spacers for all 130 basic units. In 1999 a domestically produced valved spacer was available and deemed by the teams better adapted to the local needs [3].

## Target Population

The program initially selected, as its target population, users of the public health system and, for their susceptibility, children younger than 5 years, who accounted for approximately 60% of all asthma-related hospitalizations in the year preceding the program. Additionally, children of this group who had been diagnosed with acute and recurrent pneumonia were also part of the target population because previous studies showed that misdiagnosis is common and wrongly given to children with asthma. This was demonstrated in another study, published by our group, in which 64.4 (95% CI, 57.3-70.9) of 202 hospitalized children were diagnosed with repetition pneumonia at hospital discharge, but after outpatient re-evaluation were reclassified as having asthma [4]. For this reason, children diagnosed with asthma and pneumonia were initially recruited. In the pilot outpatient clinic run, it was verified that 100% of the children diagnosed with repetition pneumonia actually had asthma, according to GINA criteria.

These results obtained in our setting are coherent with another historical cohort study where North American authors demonstrated that several "recurrent pneumonia" cases were, in fact, patients with asthma [5].

At an early stage, children older than 5 years and adolescents were referred to secondary care facilities, and later were gradually absorbed by the primary care facilities.

## Data Collection

In an effort to involve sectors of the Municipal Health Authority of Belo Horizonte, an information system was devised and implemented. At first, this was done manually, through a simplified medical chart that contained, essentially, patients' personal information, previous history of emergency room visits and hospitalizations, asthma classification, therapeutic scheme, and disease evolution. Beginning in 2000, the program was digitalized and an electronic medical chart was developed in a software program called ASMACAD--asthma patients control system--which generates reports on patients, dispensed medication, hospitalization and emergency room history, and the total number of patients in each facility. This helped to improve quality because the primary care facility has access to data of patients hospitalized by either asthma or pneumonia, allowing for more promptness at screening and optimization of one of the project's main objectives: reducing hospital admissions and re-admissions and emergency room visits.

## Support Material

Posters were designed to be hung permanently at each facility and to contain information related to the exacerbation treatment, quality verification of inhalation technique, asthma severity classification, prophylactic treatment, and an educational chart for parents. Each child assisted by the program receives an identification card detailing asthma severity, inhaled corticosteroid in use, recommended treatment in case of exacerbation, and referral request to the more convenient primary health center. This material is reviewed to make sure it corresponds with the current therapy and social needs.

## Educational Component

This is one of the essential strategies of the program and involves the health team, family, patients, and community. During the medical and nursing consultations, and in operative groups, aspects such as the need for the patient to be involved with the health team, to comply with the treatment, to avoid allergens, to engage in physical activity, and to recognize factors leading to exacerbations (initial manifestations and limits of their self-management ability) and signs of respiratory insufficiency are discussed, aiming to raise awareness.

Operative groups are moments where the children and their parents learn to deal with the disease and to understand that they have important roles in the treatment of asthma. They act as an important way to integrate doctor, team, and patient, and it is only effective in increasing adherence and subsequently reducing morbidity. These educative aspects are taken as part of an integral health strategy, and since the program's inception, festive events have taken place in which children can engage in sports, music, and theater, making patients, their families, and community aware that children with asthma can live their lives without restrictions.

## Ethical Aspects

The Institutional Review Board was always consulted and approved the research projects conducted in Wheezy Child Program facilities.

## Achieved Results

### Continued Medical Education

Human resources constitute the most important part of a health system, and the inexistence of a formal process of training is one of the main difficulties in the implementation of national and international guidelines for asthma. A 1999 assessment, with a sample size of 71 trained pediatricians, showed that 60% considered the content of the training adequate; 71% reported an increase in the team's sensibility to treat cases; 60% had already shared their knowledge with the nursing team; 82.3% felt able to use the inhalation technique; and 78.7% were already using the protocols for exacerbations and maintenance treatment. At the end of the training, 30.4% asked for continued education and periodic meetings, and 30% suggested that additional training should be done as soon as possible.

Analysis of this first assessment showed the success of the training, focused on practical learning and contextualized in the epidemiological scenario, and directed to a team seeking new answers and having medications and valved spacers at their disposal. A second inquiry, carried out with 124 other pediatricians, showed similar results. Eighty-six percent considered themselves able to screen patients who required inhalation therapy, and about 50% were able to follow the patients clinically as well. Furthermore, 70% of the trainees considered themselves more prepared to identify and treat acute asthma with pressurized metered dose inhalers (pMDIs) with *β*_2_-agonists, which led to a reduction in the number of referrals to hospitalization.

Between 1996 and 2000, 350 pediatricians and 250 nurses were capacitated, and 25 physicians received specialized training, becoming reference professionals in the treatment of asthma in their respective sanitary districts.

The education process carried out in 2001 in the 4 emergency departments units capacitated 71 pediatricians, 28 nurses, and 80 caregivers for the treatment of acute asthma.

### Population-Based Study: Impact in Reducing Hospitalizations

Because the reduction in the number of hospitalizations is one of the main objectives of the program, Table 1 shows their impact.

**Table 1:** Number of Hospitalized Children Before and After Admission in the Asthma Management Program in Belo Horizonte (n = 5301)

Hospitalization	Before Admission n (%)	After Admission n (%)
Yes	2149 (40.5%)	453 (8.6%)
No	3152 (59.5%)	4848 (91.4%)
Total	5301 (100%)	5301 (100%)

Of the 2149 patients with a history of hospitalization after program admission, only 453 were re-admitted in the 6 to 12 months after program admission, a reduction of 79% in frequency [6]. This rate is comparable with those obtained internationally, which showed reductions of 80% to 86%, but far from the 100% attained by Cabral et al in a study that involved 50 impoverished children from the state of São Paulo [7]. The choice of the study design, ie, a before and after trial, with known limitations, was justified by the difficulties of developing an evaluation study in a city as complex as Belo Horizonte, and it was indicated as a valid design by Gordis [8]. Nevertheless, it is common to use frequency of hospitalizations and emergency departments visits or emergency room non-scheduled visits as parameters to evaluate treatments for controlling asthma because these variables are quantifiable and are important events for patients and their relatives, which reduces recall biases.

**Figure 1:**
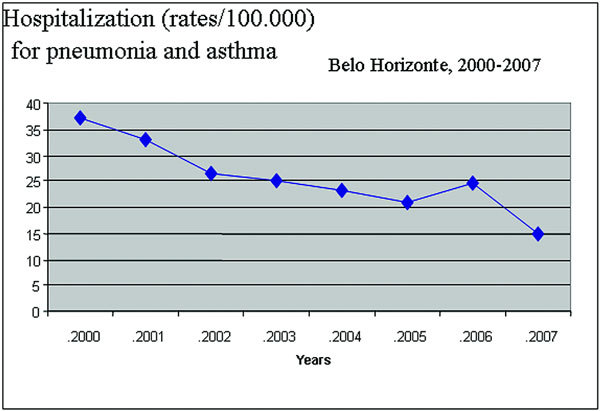
**Hospitalization rates for asthma/pneumonia: 2000-2007**.

Hospitalization rates for asthma and pneumonia are demonstrated in Figure 1 and show that asthma- and pneumonia-related hospitalizations decreased by 60% from 2000 to 2007 (*P *= 0.002) in Belo Horizonte.

### Ambulatory-Based Studies

#### Treating Exacerbations With pMDI-Valved Spacers

Aside from involving the child with the primary care facility for preventive treatment, it was also necessary to treat exacerbations with pMDIs and spacers and to leave oxygen-driven nebulizers only for treatment of severe exacerbations with hypoxemia. Therefore, emergency departments professionals were capacitated for optimal treatment of exacerbations.

**Figure 2:**
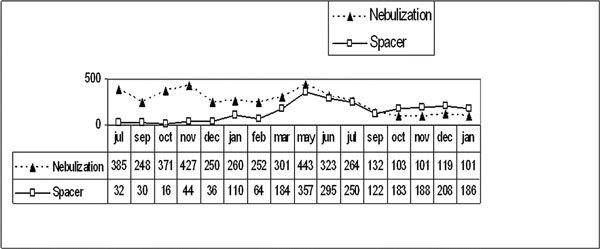
**Modality of treatment employed to treat acute asthma: absolute number of nebulizations and metered dose aerosol with spacer: from July 2000 to January 2002**.

Figure 2, obtained from the records of one of the outpatient referral clinic, shows data of the use of pMDIs-spacers and the oxygen-driven nebulizer in acute asthma. In the 9 months preceding the health team training, there were 3010 cases, 516 (17%) were treated with *β*_2_-agonists aerosol with spacer and 2494 (83%) with nebulizers. In the period after the specific training there were 3375 cases, and there was a 300% increase in the use of aerosol and a reduction to 50% in the use of oxygen-driven nebulizers (*P *< 0.001) [6]. Similar results were later obtained in other program facilities by different teams. The main reason pMDIs-spacers have currently shown sustainable achievements lies in the cost savings obtained by this treatment strategy, in which the pMDI-spacer approach was found to be 5 to 8 times less expensive than one using nebulizers.

#### Adherence Rate to Beclomethasone Diproprionate

A prospective cohort study involving 172 children and adolescents with moderate and severe asthma registered in the CSORC has shown that the median adherence rate was 50% and that many children did not attain clinical control of the disease because of nonadherence. Compliance rates reported by parents and/or guardians were always higher (*P *< 0.001) and exhibited a weak correlation with pharmacy records during the period studied: 4th (r = 0.37) and 12th (r = 0.31) month follow-ups [9].

Accordingly, it was suggested that the Municipal Health Authority and the health team should monitor the adherence rates. Examination of pharmacy records was valuable, inexpensive, and easy to perform and has been progressively implemented in the asthma program framework [9].

#### Factors Related to Lower Adherence Rates to Beclomethasone Dipropionate

A concurrent cohort study was carried out for 24 months in 168 randomly selected patients suffering from persistent moderate asthma and, again, adherence rates were verified by pharmacy records [10]. Factors associated with adherence rates < 70% in the 4th month were as follows: the mother's low schooling level (*P *= 0.03, OR = 2.25, 95% CI, 1.07-4.73), consultation attendance rate lower or equal to 2 (*P *= 0.04, OR = 2.30, 95% CI, 1.03-5.13), and prescription of more than 2 puffs per day (*P *= 0.001, OR = 2.99, 95% CI, 1.38-6.48). In the 12th month, those factors were associated with the following: mother's low schooling level (*P *= 0.04, OR = 2.21, 95% CI, 1.05-4.63), low consultation attendance rate (*P *= 0.02, OR = 2.71, 95% CI, 1.18-6.22), patient younger than 7 years (*P *= 0.037, OR = 2.23, 95% CI, 1.05-4.75), and replacement of caregiver (*P *= 0.01, OR = 8.14, 95% CI, 1.73-38.3). Finally, in the 24th month, the factors were as follows: absence of allergic rhinitis (*P *= 0.03, OR = 2.68, 95% CI, 1.08-6.61), consultation attendance rate lower than 2 (*P *= 0.04, OR = 2.29, 95% CI, 1.03-5.10), and replacement of caregiver (*P *= 0.03, OR = 2.52, 95% CI, 1.07-5.91). Only the number of consultations lower than 2 in a 4-month period was associated with a lower adherence rate in all study periods (*P *= 0.02) [7]. Results have shown that the above adherence rate determinants should be re-evaluated continuously and the health team at primary care facilities should recognize and facilitate the access of patients needing special care, which can contribute to better adherence and reduction of asthma morbidity [10].

#### Adherence Rate to Inhaled Corticosteroids and Their Impact on Asthma Control

The aim of this concurrent cohort study was to evaluate the association of adherence rates to beclomethasone diproprionate and the degree of asthma control during 1 year of follow-up. One hundred twenty-two patients with asthma, aged 3 to 12 years, were randomly selected in the CSORC. Once more, adherence rates were verified by pharmacy records and asthma control was assessed through a scoring system comprised of 4 variables (nocturnal and morning symptoms, limitation of physical activities, and exacerbations). Fewer than half (40.3% maximum) the 122 patients maintained asthma control. There were statistically significant differences in adherence rates for maintaining or not maintaining the asthma control and optimal asthma control entailed adherence rate higher than 80% [11].

#### Risk Factors for Emergency Room Visits

A cross-sectional study demonstrated that, in patients enrolled in the program with moderate and severe persistent asthma, prevalence of allergic rhinitis was of 74.6% (95% CI, 65.9-81.7). The presence of allergic rhinitis (OR = 2.98, 95% CI, 1.10-8.06) and asthma severity (OR = 2.09, 95% CI, 1.05-4.44) were independent risk factors for nonscheduled emergency room visits because of acute asthma. Accordingly, the Municipal Health Authority recommended to the primary health care facilities team to make all efforts for an early diagnosis and treatment of allergic rhinitis in asthmatic patients assisted by the asthma program [12].

#### Allergic Rhinitis

Phase III of the International Study of Asthma and Allergies in Childhood showed prevalence of allergic rhinitis in 27.9% of patients in Belo Horizonte [13]. Studies employing standardized and validated questionnaires reported rhinitis in 98.9% of atopic asthmatic patients and 78.4% in nonatopic patients [14]. Two studies were carried out with children and adolescents with moderate and severe persistent asthma for the Wheezy Child Program and showed that inhaled nasal corticosteroids were effective in the simultaneous treatment of asthma and allergic rhinitis [15,16].

#### Program Expands: Target Population Is Now Adolescent

A survey conducted in the Belo Horizonte public schools with adolescents who had asthma revealed that they presented emotional and behavioral difficulties not approached in the standardized asthma protocols [17]. We felt that the factors should be accounted for in the Wheezy Child Program.

#### Prevalence of Wheezing in Infants in Belo Horizonte

Belo Horizonte took part in the International Study of Sibilance in Infants and among 1261 infants (aged 12 to 15 months), randomly selected, there was a 52% prevalence of sibilance. Assistance in emergency services occurred in 63.2% of cases, and infants were hospitalized with recurrent wheezing in 70.1% of the cases. These findings confirm the high prevalence and the associated morbidity [18].

Data from Belo Horizonte are consistent with those found in Curitiba, where the prevalence of sibilance was 45.4% [19]. Childhood sibilance has been widely studied, especially its relation to viral infections and therapeutic strategies [20].

## Final Remarks

The Wheezy Child Program, with 30,000 children enrolled, was the first of its kind in Brazil and it has shown its viability in any municipality, sustainability, and high social and economic impact.

The program has significantly contributed to the qualification of human resources and to scientific production with monographs, theses, and dissertations that bring new and relevant information and point to directions the program should head.

The implementation of a program continuously creates new demands. In the past, there was the need for inhaled steroids, and now we are concerned with fighting adherence rate to maintain treatment and failures in controlling asthma. Knowledge about asthma has increased, and the standardization of new drugs for handling the most complex cases must be considered.

It is estimated that, among the children and adolescents in the program, 2500 have problems in controlling asthma and have demanded individualized attention from the Wheezy Child Program. Thus, the technical cooperation between UFMG and the municipality of Belo Horizonte was resumed.

The high prevalence of and morbidity among sibilant infants in Belo Horizonte, which remained unaltered since the beginning of the program, creates the need to identify among them those who also have asthma and would benefit from the therapeutic strategies in the program (inhaled corticosteroids).

Additionally, a multidisciplinary approach is needed when treating adolescents, to better help them contend with the emotional difficulties prompted by years of preventive treatment.

The population of Belo Horizonte has increased 23% in the last 15 years, and the program needs to continuously adapt to provide for this new population. The context of human resources shifted in 2001, when the assistance model began to be directed by the Family Health Program, and the training strategies have changed accordingly.

As of 2009, the Wheezy Child Program completes 15 years in the primary care of Belo Horizonte, with plenty to celebrate and with new challenges ahead. These challenges must be seen as encouragement to improve and keep the program alive and relevant.
